# Primary Hepatic Melanoma: A Case Report

**DOI:** 10.7759/cureus.105195

**Published:** 2026-03-13

**Authors:** Mohammed Bakhti, Chaimae Bekhakh, Anass Haloui, Nassira Karich, Amal Bennani

**Affiliations:** 1 Department of Pathology, Mohammed VI University Hospital, Oujda, MAR; 2 Faculty of Medicine and Pharmacy, Mohammed I University of Oujda, Oujda, MAR

**Keywords:** immunohistochemical, liver, melanoma, primary, undifferentiated

## Abstract

Primary melanoma of the liver is very rare, with only a few cases reported. We present a 69-year-old patient with no significant medical history, admitted for the discovery of hepatic nodular lesions whose radiological examination suggested hepatocellular carcinoma. The biopsy of the nodular lesion revealed an undifferentiated malignant tumor, and immunohistochemical analysis using anti-SOX10, anti-Melan A, and anti-HMB45 antibodies confirmed melanocytic origin. Subsequently, the patient underwent additional examinations to search for the primary site, which were negative. Ultimately, the diagnosis of a primary melanoma of the liver was established.

## Introduction

Primary melanoma of the liver is an extremely rare malignant tumor, with only a limited number of cases documented in the literature [1]. The diagnosis of primary hepatic melanoma presents challenges due to its ambiguous clinical manifestations and nonspecific radiological characteristics [2]. First, a hepatic metastasis from a melanoma must be ruled out. This occurs relatively frequently, in 10% to 20% of cases, and is associated with a poor prognosis as well as limited survival [3]. In this report, we present a case of a patient whose histological examination confirmed the presence of a primary liver melanoma.

## Case presentation

**Figure 1:**
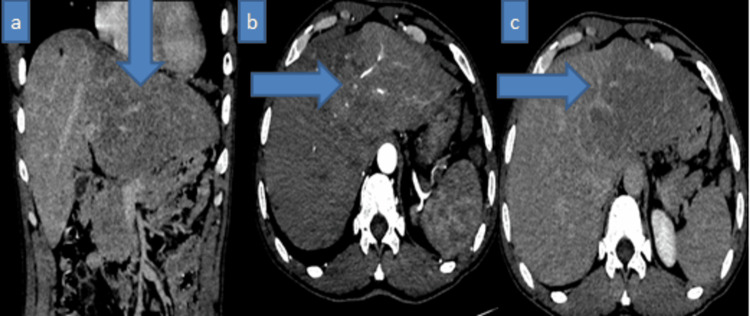
Enhanced abdominopelvic CT scan in coronal (a) and axial (b, c) views showing a hypodense area involving the left liver (a), with enhancement in the arterial phase (b) and washout in the late phase (c), causing invasion of the left portal branch of hepatic segment II. Blue arrows indicate the tumor lesion.

**Figure 2:**
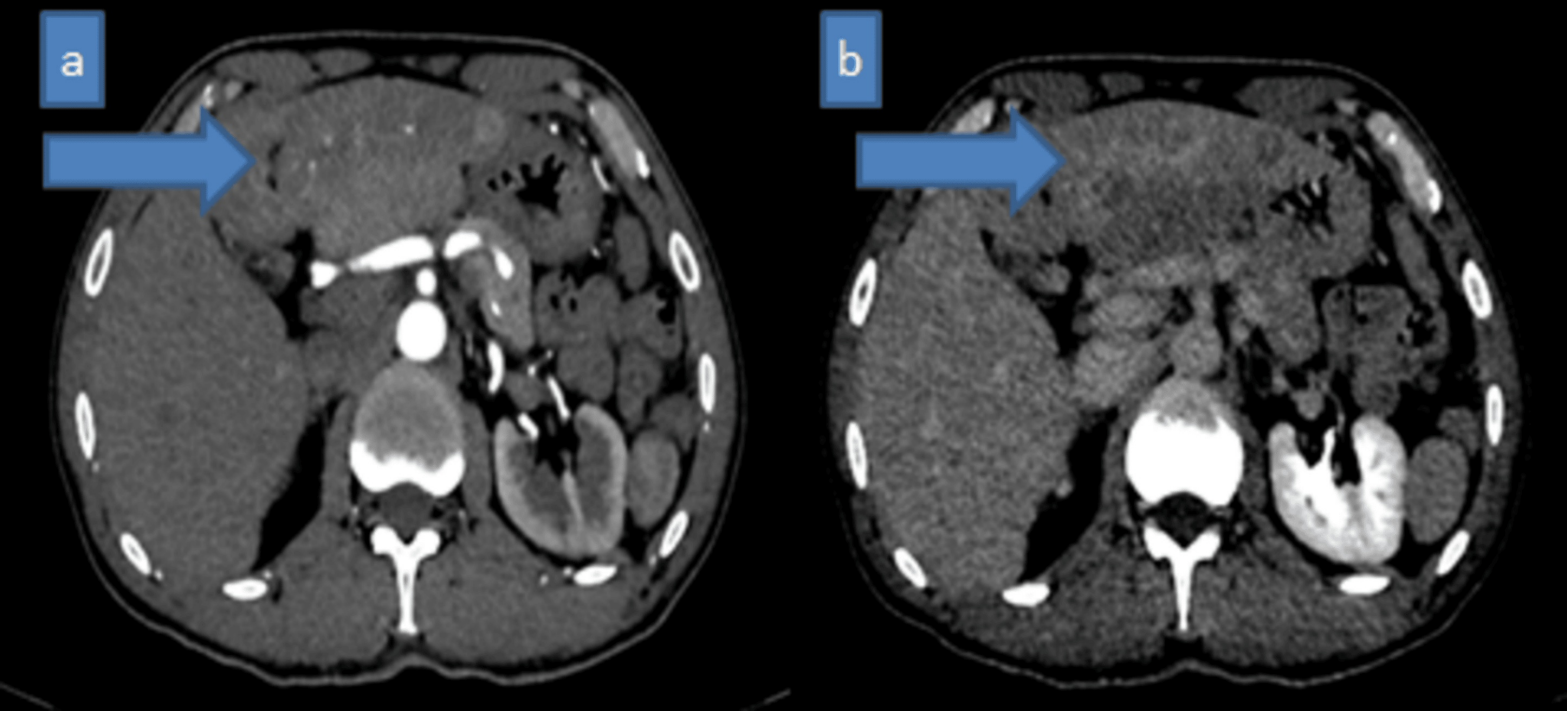
Axial CT images in the arterial (a) and late (b) phases showing several nodular daughter lesions (blue arrows) within the hypodense area, hypervascularized in the arterial phase (a) with washout in the late phase (b). These scanographic findings are suggestive of infiltrating hepatocellular carcinoma.

A 69-year-old man, without notable personal or family history and no history of alcoholism or infection with hepatitis B or C virus, was admitted to the visceral surgery department for the incidental discovery of multiple nodular lesions on CT scan initially suggesting hepatocellular carcinoma (Figures 1-2). The management plan was to perform a percutaneous biopsy of the lesion under ultrasound guidance and send it to our pathology department for histological confirmation.

**Figure 3:**
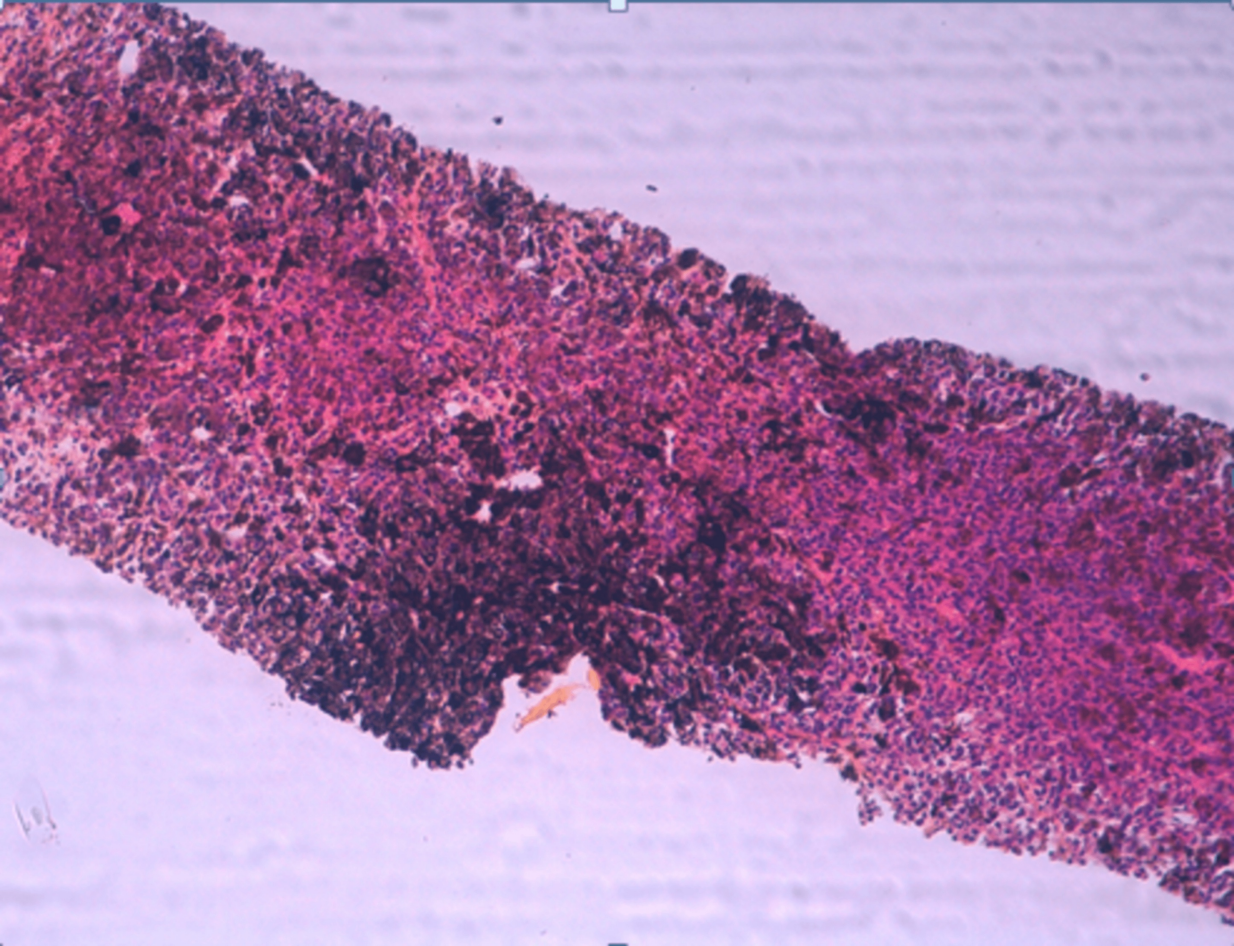
Biopsy core showing malignant tumor proliferation (low magnification).

**Figure 4:**
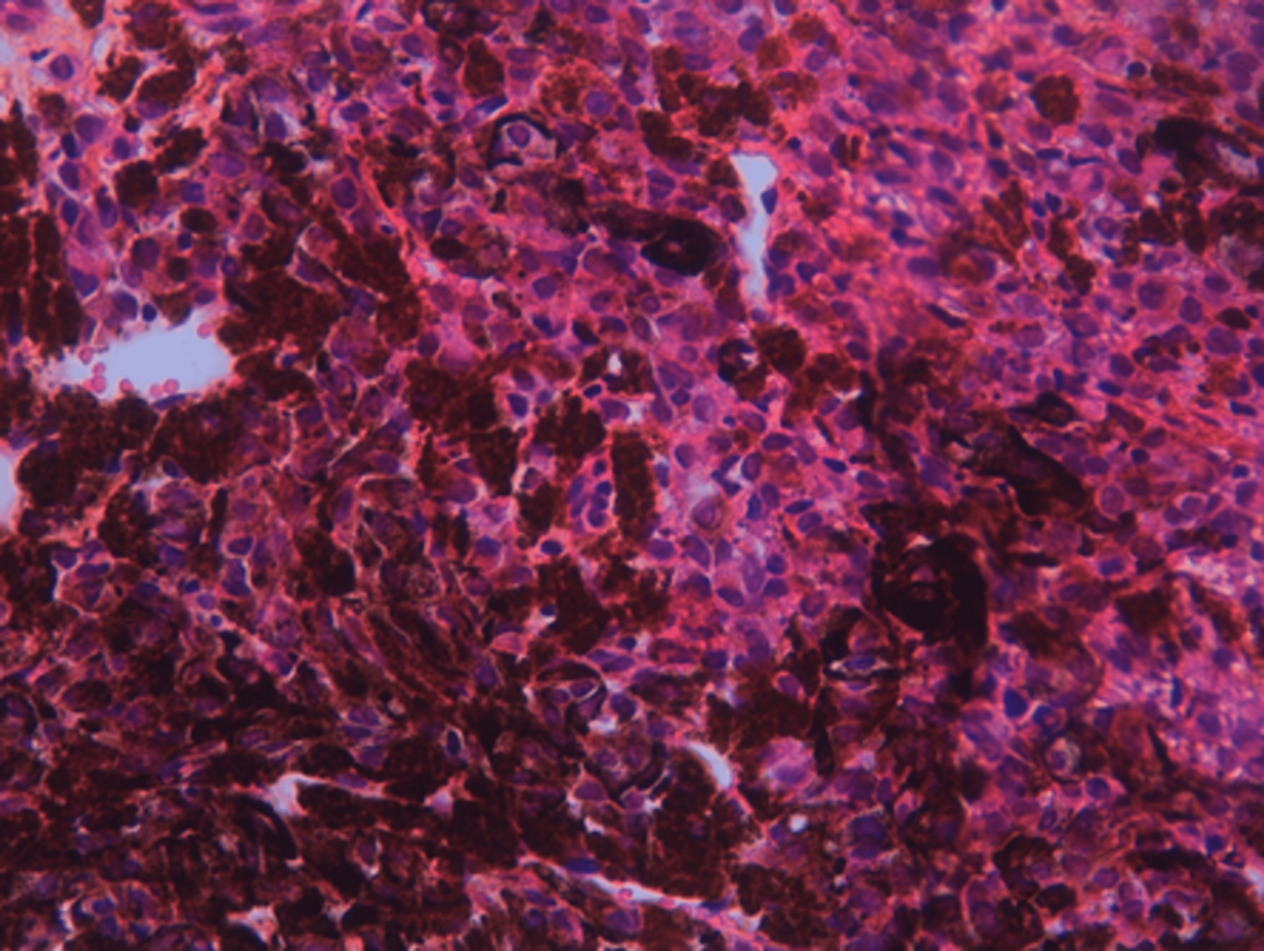
Malignant tumor proliferation composed of atypical cells with brownish pigment (high magnification).

Histological examination revealed liver parenchyma extensively infiltrated by a tumor proliferation with diffuse architecture, composed of frankly atypical cells featuring large nuclei with prominent nucleoli, abundant eosinophilic cytoplasm, and brownish pigments (Figures 3-4).

**Figure 5:**
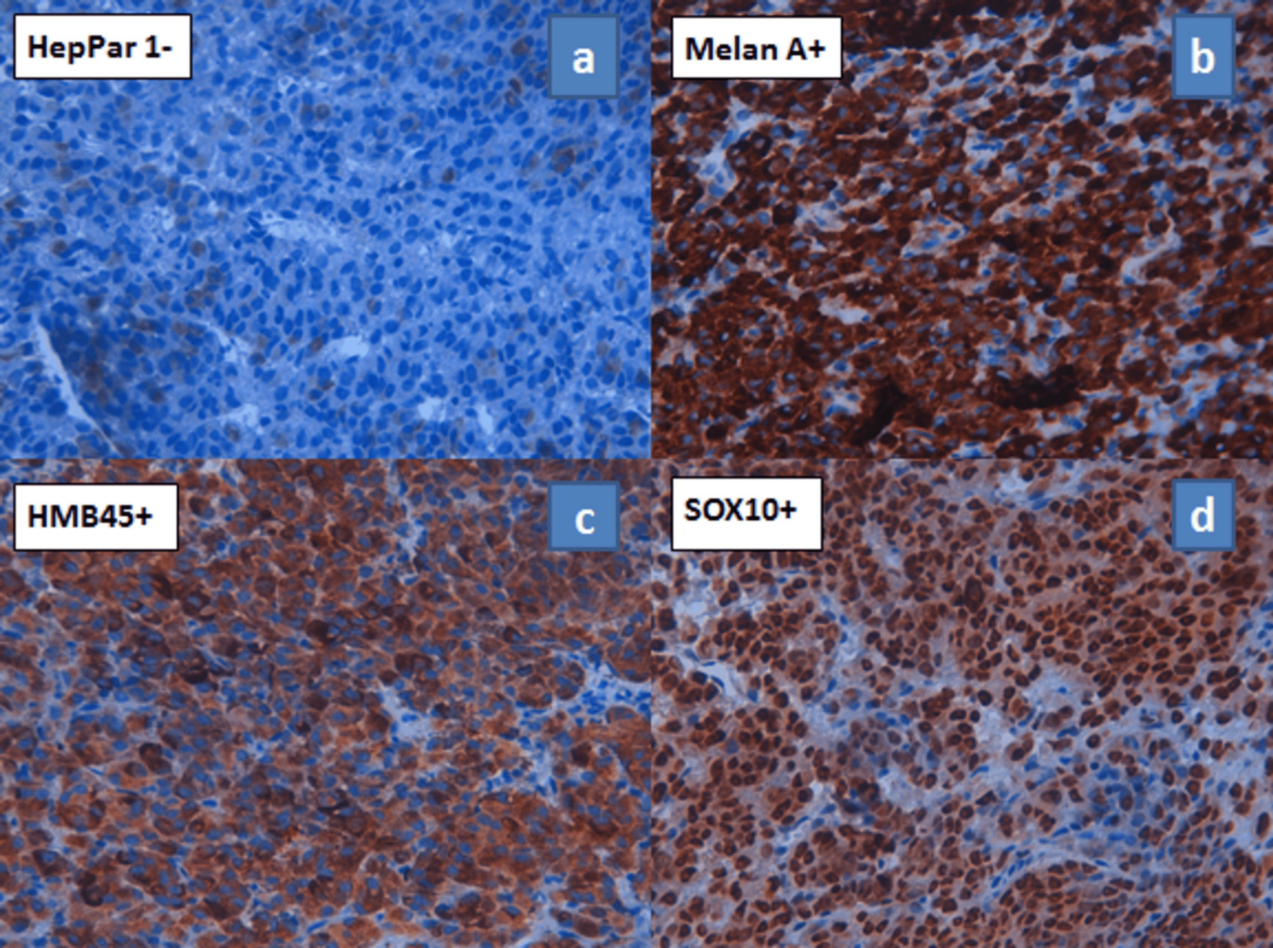
Immunohistochemical study showing absence of staining for the anti-HepPar1 antibody (a) and positive staining for anti-Melan A, anti-HMB45, and anti-SOX10 antibodies (b, c, d) (high magnification).

An immunohistochemical study was performed, which showed diffuse positive staining of tumor cells with anti-Melan A, anti-SOX10, and anti-HMB45 antibodies, and the absence of staining with anti-HepPar1 antibody (Figure 5).

The diagnosis was hepatic localization of a poorly differentiated tumor process with an immunohistochemical profile compatible with melanoma. Subsequently, the patient underwent dermatological, anogenital, ophthalmologic, and endoscopic examinations to search for a primary pigmented lesion, all of which were negative. Therefore, a final diagnosis of primary hepatic melanoma was established.

## Discussion

Primary melanoma of the liver is an extremely rare, nonepithelial malignant tumor. It has not been sufficiently described, and only two cases have been reported in the literature [4,5].

The pathophysiology of primary liver melanoma is not well understood, and several hypotheses have been suggested. The most widely supported hypothesis is the regression of the primary site following an immunological response by the human body, since the liver does not contain melanocytes. Some authors believe that this tumor develops in the liver following a malignant transformation of ectopic melanocytes [6,7]. Alternatively, it could be due to the malignant transformation of melanoblastic cells from the neural crest during embryogenesis [2].

The diagnosis of primary hepatic melanoma rests on fulfilling three key criteria. First, confirmation is obtained through histological and immunohistochemical studies that identify characteristic melanocytic markers. Second, it requires the exclusion of any other primary melanoma site through comprehensive imaging and clinical evaluation. Third, there must be no history of a cutaneous lesion, particularly one that was resected without histological proof [1].

Clinically, the symptomatology is variable and nonspecific, characterized mainly by an alteration of the general state of health, a mass, or abdominal pain [8]. CT scanning is the most commonly used examination that allows for the precise determination of the site and appearance of hepatic tumor proliferation [1].

Histologically, primary melanoma of the liver is characterized by a variable morphological appearance, where the cells can take on an epithelioid or spindle-shaped aspect, with or without melanin deposition. Several mitotic figures have been noted. For difficult amelanotic cases, an immunohistochemical complement is highly recommended [9].

Surgery associated with lymphadenectomy of the hepatoduodenal ligament constitutes the best therapeutic approach for well-defined and resectable forms [1]. Interferon-based immunotherapy can reduce the risk of recurrence [10]. Very limited efficacy has been shown by dacarbazine-based chemotherapy [11].

The prognosis is poor and even worse than other types of melanoma, likely due to diagnostic delay and its very rapid evolution [4,5,12-14].

## Conclusions

Primary melanoma of the liver remains an extremely rare clinical entity, posing a significant challenge for both oncologists and pathologists. Its diagnosis requires the systematic exclusion of any other primary site, often necessitating extensive investigations. This rarity is a major obstacle to establishing standardized therapeutic protocols. Despite recent advances in immunotherapy, the overall prognosis remains poor.
